# From Art to Engineering? The Rise of In Vivo Mammalian Electrophysiology via Genetically Targeted Labeling and Nonlinear Imaging

**DOI:** 10.1371/journal.pbio.0030355

**Published:** 2005-10-11

**Authors:** David Kleinfeld, Oliver Griesbeck

## Abstract

A convergence of technical advancements in neuroscience has begun to transform mammalian electrophysiology from an art into a precise practice.

For close to half a century, neurophysiologists have been able to record electrical signals from the millions of individual neurons that compose even the smallest mammalian brain. Despite this long history, which has led to significant strides toward understanding how neuronal activity translates into brain function, much of the way electrophysiological data is gathered is more of an art form than a science. Reproducibility, a cornerstone of scientific progress, hasn't always been forthcoming when recording from individual neurons in the brain, largely because of the improbability or uncertainty that different investigators make their measurements from the same neurons or even the same select subpopulations of neurons. How does one put together the information about activity in single neurons that are recorded by different investigators in different ways? Further still, how does one combine this information with knowledge of the underlying circuitry to make sense of the firing patterns that underlie normal brain function? Progress will come largely from the ability to reproducibly record voltages, as well as other variables that define physiological function, from identified neuronal cell types. The ability to record from the same subpopulation of cells on a routine basis is the singular means to validate measurements across different laboratories and move electrophysiology beyond its current, largely anecdotal status.

By definition, measurements from identified neuronal cell types depend on a means to visualize the cells in question. For simple neuronal circuits in invertebrates, in which the function of a cell is often well correlated with its physical location within a ganglion, simple light microscopy imaging is adequate to uniquely identify a neuron. Similarly, in a mammalian brain slice, gross architectonic features can be discerned from the visual texture of the tissue, while individual neuronal boundaries may be identified with optical techniques that minimize the interfering effects of scattered light. For the case of recording from brains in living mammals, the technical challenges that must be surmounted to record from identified cells are far greater. Antidromic activation of projection neurons, a heroic approach, provides selectivity in some instances [1]. Yet, at present, much of in vivo recording is performed blind, in the sense that cell morphology and phenotype are confirmed only from post hoc histology.

What advances lie ahead to advance the qualitative nature of mammalian in vivo recording? In particular, can electrophysiology approach the level of precision and reproducibility that one associates, for example, with biochemistry or molecular biology? The confluence of three avenues of technical advance—one in imaging, one in labeling, and one in behavioral training—suggest that in vivo electrical and optical recording from identified neuronal phenotypes in the central nervous system of awake behaving mice should soon be a common reality. That's the good news. But before we get too enthusiastic, it is important to realize that the major stumbling block in electrophysiology has yet to be solved. Electrophysiology remains a labor-intensive art form. Data gathering involves many manually controlled processes that require an extended and constant level of vigilance. This is to be contrasted with molecular biology, where standard tools and high levels of automation make acquisition relatively cheap in terms of time and expense, and thus shift the focus to conceptual synthesis. Time will tell if the technical advances described below advance not just the reliability of electrophysiology but further serve as a tipping point for its transition from an art to an engineering process.

## Labeling of Specific Neuronal Phenotypes—Mus musculus as a “Simple” Nervous System

The age of transgenic animals and fluorescence labeling drives forward with ever greater abandon. Neurobiology is one of the great beneficiaries of the development of a rainbow spectrum of fluorescent proteins (XFPs) [2–4], in the sense that transgenic expression of these proteins reveals the three-dimensional outlines of individual living neurons with minimal cytosolic perturbation. For the electrophysiologist, this portends the engineering of mice in which defined subclasses of neurons express a fluorescence label. While this technique in mammals is not quite at the level of precision it has in invertebrates, where one can often identify individual neurons in vivo, such mice offer the possibility of allowing researchers to return to the same phenotypically defined neurons within a given brain region. A demonstration of the power of this approach is a well appreciated series of transgenic mice labeled via nonhomologous incorporation of an expression cassette (a short sequence of DNA) that codes for the pan-neuronally expressed Thy-1 promoter, a selected XFP, and ribosome binding [5]. The type of expression varies significantly from line to line as a result of strong positional and context sensitivity of the Thy-1 expression cassette when integrated into the genome. The resultant mosaic labeling is valuable for certain studies, but more importantly, it serves to illustrate that considerable “artistic” elements are currently at work in labeling the brain.

**Figure 1:**
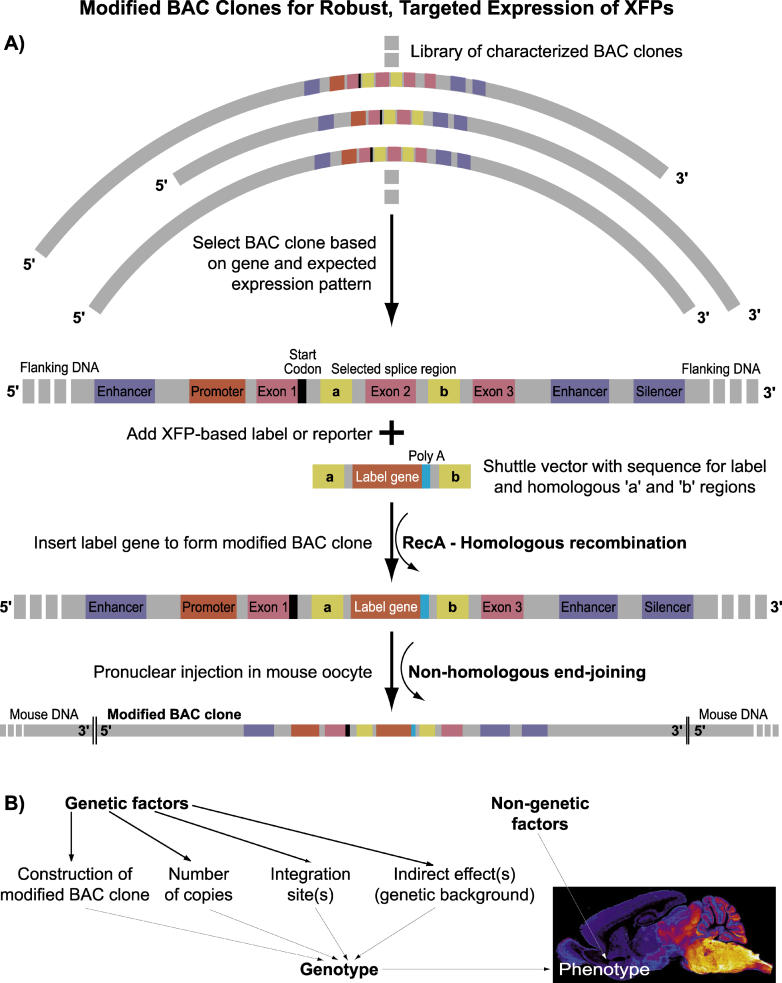
Schematic for the Production of a Modified BAC for the Targeted Expression of an XFP or an XFP-Based Reporter in Mice (A) A library of suitable BAC clones is scanned using bioinformatics and an appropriate clone, encoding a suitable cell-type-specific transcriptional unit with ample flanking regions, is selected. Note that only a few of the many possible enhancer and silencer regions are drawn. An exon that lies downstream of the ATC start sequence is selected to be replaced by the XFP/reporter sequence by homologous recombination (exon 2 in this example), and a shuttle vector that codes for the label together with flanking regions around the exon (“a” and “b”) is constructed. The enzyme RecA is used to interchange the sequence for the exon and the label to form a modified BAC clone that codes for the label. The modified clone is injected into a mouse oocyte, where the dominant incorporation into the host DNA occurs through nonhomologous recombination. (B) Many factors influence the phenotype of a given transgenic mouse, and thus the same clone may result in a number of lines with slightly different properties. The insert shows the XFP expression pattern for a line based on a BAC clone that contains the transcriptional unit of a glycine transporter. (Image: Jean-Marc Fritschy and Hanns-Ulrich Zeilhofer)

What are the essential difficulties in reproducibility and predictability in the generation of mice with labeled neurons? The use of expression cassettes in mammals suffers from the difficulty of identifying key regulatory elements, such as enhancers or silencers, that are necessary for the correct expression of a transgene [6]. A related source of variability is that expression of the label is influenced by the DNA sequences that flank the inserted DNA, yet the site of integration into the genome differs between transgenic animals. These difficulties are diminished through the use of bacterial artificial chromosomes (BACs) [7,8], which incorporate the entire transcription unit and large pieces of sequence 5′ and 3′ of it (Figure 1A). Although this approach is not perfect and can still miss out on important regulatory elements in some cases (Figure 1B), in the ideal case the BAC includes all necessary elements to express a reporter gene in the correct manner.

Methods to incorporate reporter genes into BAC constructs are relatively straightforward (Figure 1A) and have led to an almost industrial-scale effort to generate and characterize a collection of mice with defined labeled neurons for further anatomical and physiological analysis [9]. Recent examples of transgenic mouse technology based on BAC clones demonstrate the accurate labeling of neurons containing the neurotransmitter glycine in the spinal cord, brainstem, and cerebellum (Figure 1B) [10], and the labeling of neurons expressing both parvalbumin and GABA throughout neocortex [11]. Other examples used clones with the gene for glutamic acid dehydrogenase (GAD-27) to select for all GABAergic neurons, but observed expression in only the parvalbumin-positive subpopulation [12,13]. It is to be expected that the precision of molecular biology will further evolve to produce mice with ever increasing specificity of subtype labeling.

## In Vivo Visually Guided Recording of Labeled Cortical Neurons—Laser Jocks Turned Neuroscientists

**Figure 2:**
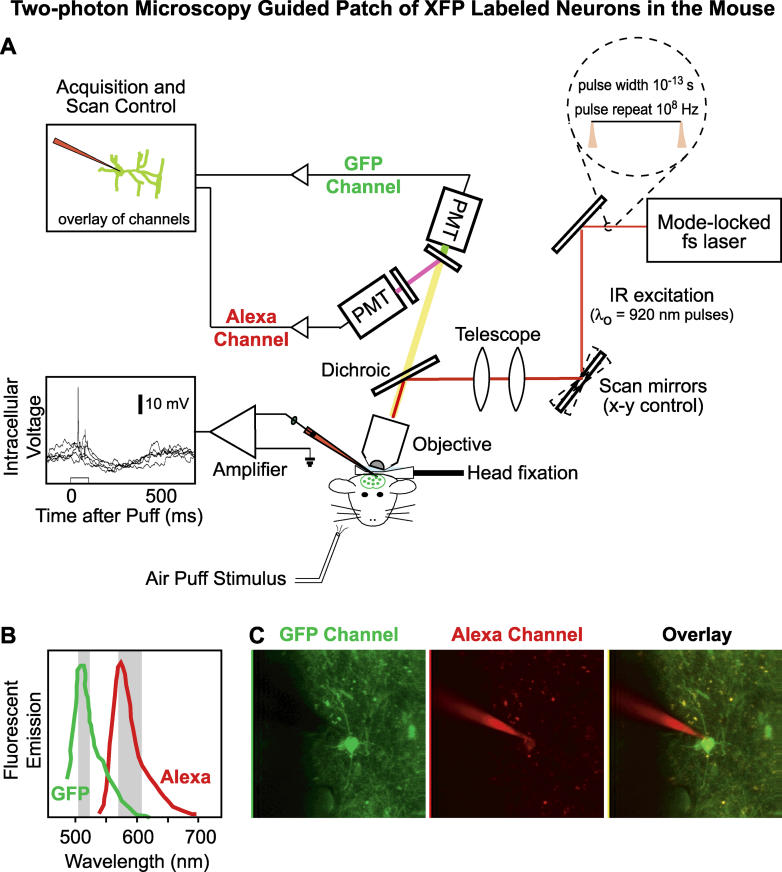
Targeted Electrical Recording of Transgenically Labeled Inhibitory Interneurons in Mouse Cortex (A) The two-photon laser scanning microscope is shown schematically. The critical features are the use of separate fluorophores, one for the label (GFP in this example) and another to mark the intracellular fluid of electrode (Alexa in this example) that have overlapping excitation spectra and different emission spectra (see [B]). The intracellular voltage shows a trace obtained under whole-cell patch of the response to vibrissa stimulation. Alexa, Alexa 594 dye; fs laser, titanium:sapphire mode-locked laser with 100 fs output pulse width; GFP, green fluorescent protein; PMT, photomultiplier tube. (B and C) Emission spectra and fluorescent images from the GFP and Alexa channels. Confirmation of whole-cell patch is achieved by injecting Alexa into the GFP-filled cell, as illustrated in the overlay. (Images: Troy Margrie)

Making mice with fluorescent neurons is only the first step; the second requires the means to visualize the axons and dendrites of these neurons, which can be less than a micrometer in thickness. In vivo two-photon laser scanning microscopy (TPLSM) [14,15] provides a unique means to image fluorescently labeled neurons that lie below the surface of the brain [16]. When used in conjunction with transgenic mice that are labeled by the expression of a fluorescent protein, TPLSM provides the necessary visualization to target a fine glass electrode to the membrane surface of one's neuron of choice [17] (Figure 2). TPLSM can image deep into scattering brain tissue, in excess of 500 μm under normal conditions [18] and down to 1,000 μm under special circumstances [19]. Although technical challenges—such as increasing the rate at which images are scanned and compensating for optical aberrations—one can, in principle, image and thus target neurons throughout almost the entire depth of mouse cortex.

## Biomolecular Reporters and Drivers of State Variables—Proteins as Spies and Membrane Provocateurs

Apart from their role as phenomenally good, noninvasive labels of neuronal structure, XFPs have become the basis for a series of sensors of physiological variables and events, such as membrane-potential fluctuations and intracellular messenger dynamics [20]. Genetically encoded, these sensors are generated inside cells, do not require cofactors, and do not leak out of cells even during prolonged studies. These sensors will benefit considerably from the increasing accuracy of neuronal labeling via modified BAC clones (see Figure 1). Indicators of synaptic release [21–23] or intracellular [Ca^2+^] dynamics [24–28] might initially be the most appealing. While issues, such as signal strength and response kinetics, have still to be sorted out, recent work on transgenic mice that express these and other probes proved the feasibility of the approach (Table 1). An exquisite example is the expression of the pH indicator synaptopHluorin in olfactory sensory neurons of the mouse, which allowed for the in vivo imaging of patterns of activation in the olfactory bulb after odorant stimulation [21]. To the extent that optical microscopy is able to resolve their pattern of expression, XFP-based molecular probes offer a means to read out activity not only from a few but ideally from whole populations of identified neurons.

**Table 1:**
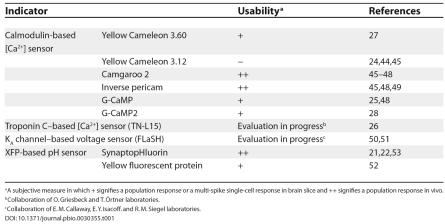
XFP-Based Indicators Expressed and Tested in Transgenic Mice

^a^ A subjective measure in which + signifies a population response or a multi-spike single-cell response in brain slice and ++ signifies a population response in vivo.

^b^ Collaboration of O. Griesbeck and T. Örtner laboratories.

^c^ Collaboration of E. M. Callaway, E. Y. Isacoff. and R. M. Siegel laboratories.

The complement to optical-based probes of neuronal state variables is optical-based perturbation mediated by intrinsic chromophores. The ability to perturb the state of neuronal activation plays two essential roles in systems identification. The first is to determine the effect of a depolarizing perturbation in neighboring as well as downstream cells. The challenge has been met, in non-mammalian systems, through the use of cloned photoreceptor complexes [29] and photolabile organic cages that release agonists of excitatory neurotransmission onto cloned channels that are expressed in defined phenotypes [30]. The second role is the inactivation of neuronal pathways as a means to open feedback loops and determine the direction of signal flow. For example, a modified K^+^ channel in which photoisomerization drives the reversible transition between closed and conducting states has been demonstrated in vitro [31]. One clear challenge is the functional incorporation of these and related photo-activated agents in defined mammalian cells.

## Targeted Recording from the Awake Rodent—Molecular Biology Meets Consciousness

**Figure 3:**
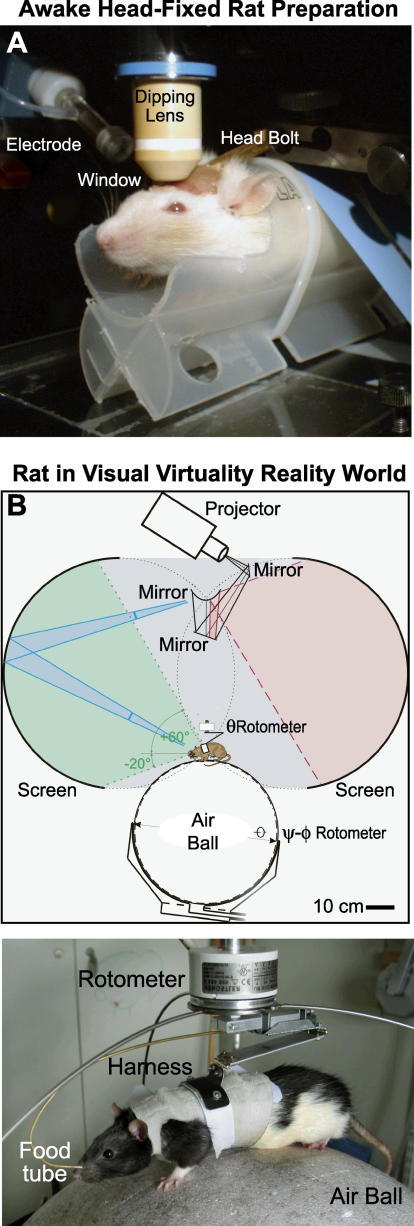
Prospects for Recording from Awake but Head-Restrained Animals (A) Photograph of a trained rat that is awake and head-restrained, ready for imaging of organic [Ca^2+^]-sensitive dyes. All aspects of the recording procedure demonstrated in primates are expected hold for mice as well. (Image: Jack Waters) (B) Photograph and set-up of visual virtual reality for rodents. In this example, the rat is body-fixed, and can rotate on an axis, but is not head-fixed. The visual world of the animal is controlled by projected images, and reward is administered through a food tube. (Images: Hansjuergen Dahmen)

Immobilization is generally necessary for most forms of recording. Needless to say, immobilization achieved by anesthesia blatantly disrupts neural function, and the whole notion of attentive-based activation as well as motor output per se is lost. This problem is avoided with primates through the use of “head-fixed” animals that are trained to sit quietly while they perceive the world through arrays of projectors and tactile pads. The same form of constraint can be brought to studies with rodents through the use of head-fixed preparations [32], which has proved to be of critical importance for the study of behavioral [33] and electrophysiological [34] aspects of whisking. This strategy has also provided a means to record both optically and electrically from individual neurons that are labeled with organic [Ca^2+^] indicators (Figure 3A; J. Waters and F. Helmchen, unpublished data), and it is anticipated that recording and perturbation from cells labeled via viral transfection will soon be forthcoming [35]. The near-term challenge is to record from awake head-fixed mice.

A final issue concerns the extent of behavior that may be expected with head-fixed animals, especially as a large block of research concerns spatial tasks and hippocampal function. Both primate electrophysiological studies [36] and human psychophysical studies [37] have advanced with the use of virtual reality. Recently, the same level of sophistication has been brought to bear on rodent studies [38] (Figure 3B), where body-fixed rats are constrained to walk on a near frictionless ball while they observe a virtual visual world. This advance already provides a means to record from rats when the tether, such as that for a head-mounted scanner [39], is too short for use with animals in mazes. In the best of worlds, this advance is a stepping stone to recording from head-fixed mice as they respond to novel environments.

## Putting It All Together

The tools are there to perform targeted electrical and optical-based ion recording, and stimulation, of identified neuronal phenotypes in mice. Nonlinear microscopy, while still a tool of the aficionado, is approaching maturity [40]. The design of endogenous molecular sensors of cell function, while in early days, has attained a set of heuristics and material successes (Table 1). This suggests that signaling and circuitry in the mammalian nervous system may be addressed in a reliable and logical, if painstaking, way. Other recent work, involving methods to automate histology at the synaptic [41] and cellular [42] levels, will help place physiological measurements in the framework of detailed architectonics. The greatest challenges for in vivo electrophysiology appear to lie primarily in the areas of molecular biology and behavior. Gene expression through the use of BACs has been successfully targeted to only a few neuronal subtypes so far, yet must be pushed to all cell types. This highlights a need for better phenotyping of neurons, both by conventional histochemistry and by microarray analysis of gene expression, and a better understanding of the transcription factor logic that defines expression. Automated means for shaping animal behavior need to be advanced [43]. Critically, while the bias that “mice cannot be trained” is pervasive, there has been little concerted effort to breed and train calm mice that could be the background for transgenesis. Behavioral issues aside, it is a good bet that a mixture of genetics and optics will play a dominant role in delimiting the algorithms of brain function.

## Abbreviations

BAC: bacterial artificial chromosome
TPLSM: two-photon laser scanning microscopy
XFP: fluorescent protein

## References

[pbio-0030355-b1] Swadlow HA, Waxman SG, Rosene DL (1978). Latency variability and the identification of antodromically activated neurons in mammalian brain. Exp Brain Res.

[pbio-0030355-b2] Prasher DC, Eckenrode VK, Ward WW, Prendergast FG, Cormier MJ (1992). Primary structure of the Aequorea victoria green-fluorescent protein. Gene.

[pbio-0030355-b3] Tsien RY (1998). The green fluorescent protein. Annu Rev Biochem.

[pbio-0030355-b4] Shaner NC, Campbell RE, Steinbach PA, Giepmans BN, Palmer AE (2004). Improved monomeric red, orange and yellow fluorescent proteins derived from Discosoma sp. red fluorescent protein. Nat Biotechnol.

[pbio-0030355-b5] Feng G, Mellor RH, Bernstein M, Keller-Peck C, Nguyen QT (2000). Imaging neuronal subsets in transgenic mice expressing multiple spectral variants of GFP. Neuron.

[pbio-0030355-b6] Rulicke T, Hubscher U (2000). Germ line transformation of mammals by pronuclear microinjection. Exp Physiol.

[pbio-0030355-b7] Yang XW, Model P, Heintz N (1997). Homologous recombination based modification in Escherichia coli and germline transmission in artificial mice of a bacterial artificial chromosome. Nat Biotechnol.

[pbio-0030355-b8] Gong S, Zheng1 C, Doughty ML, Losos K, Didkovsky N (2003). A gene expression atlas of the central nervous system based on bacterial artificial chromosomes. Nature.

[pbio-0030355-b9] Hatten ME, Heintz N (2005). Large-scale genomic approaches to brain development and circuitry. Annu Rev Neurosci.

[pbio-0030355-b10] Zeilhofer HU, Studler B, Arabadzisz D, Schweizer C, Ahmadi S (2005). Glycinergic neurons expressing enhanced green fluorescent protein in bacterial artificial chromosome transgenic mice. J Comp Neurol.

[pbio-0030355-b11] Meyer AH, Kartona I, Blatow M, Rozov A, Monyer H (2002). In vivo labeling of parvalbumin-positive interneurons and analysis of electrical coupling in identified neurons. J Neurosci.

[pbio-0030355-b12] Ango F, di Cristo G, Higashiyama H, Bennett V, Wu P (2004). Ankyrin-based subcellular gradient of neurofascin, an immunoglobulin family protein, directs GABAergic innervation at Purkinje axon initial segment. Cell.

[pbio-0030355-b13] Chattopadhyaya B, Cristo GD, Higashiyama H, Knott GW, Kuhlman SJ (2004). Experience and activity-dependent maturation of perisomatic GABAergic innervation in primary visual cortex during a postnatal critical period. J Neurosci.

[pbio-0030355-b14] Denk W, Delaney KR, Kleinfeld D, Strowbridge B, Tank DW (1994). Anatomical and functional imaging of neurons and circuits using two photon laser scanning microscopy. J Neurosci Methods.

[pbio-0030355-b15] Svoboda K, Denk W, Kleinfeld D, Tank DW (1997). In vivo dendritic calcium dynamics in neocortical pyramidal neurons. Nature.

[pbio-0030355-b16] Denk W, Svoboda K (1997). Photon upmanship: Why multiphoton imaging is more than a gimmick. Neuron.

[pbio-0030355-b17] Margrie TW, Meyer AH, Caputi A, Monyer H, Hasan MT (2003). Targeted whole-cell recordings in the mammalian brain in vivo. Neuron.

[pbio-0030355-b18] Oheim M, Beaurepaire E, Chaigneau E, Mertz J, Charpak S (2001). Two-photon microscopy in brain tissue: Parameters influencing the imaging depth. J Neurosci Methods.

[pbio-0030355-b19] Theer P, Hasan MT, Denk W (2003). Two-photon imaging to a depth of 1000 micron in living brains by use of a Ti: Al2O3 regenerative amplifier. Opt Lett.

[pbio-0030355-b20] Tsien RY (2003). Imagining imaging's future.

[pbio-0030355-b21] Bozza T, McGann JP, Mombaerts P, Wachowiak M (2004). In vivo imaging of neuronal activity by targeted expression of a genetically encoded probe in the mouse. Neuron.

[pbio-0030355-b22] Li Z, Burrone J, Tyler WJ, Hartman KN, Albeanu DF (2005). Synaptic vesicle recycling studied in transgenic mice expressing synaptopHluorin. Proc Natl Acad Sci U S A.

[pbio-0030355-b23] Okumoto S, Looger LL, Micheva KD, Reimer RJ, Smith SJ (2005). Detection of glutamate release from neurons by genetically encoded surface-displayed FRET nanosensors. Proc Natl Acad Sci U S A.

[pbio-0030355-b24] Miyawaki A, Griesbeck O, Heim R, Tsien RY (1999). Dynamic and quantitative Ca^2+^ measurements using improved cameleons. Proc Natl Acad Sci U S A.

[pbio-0030355-b25] Nakai J, Ohkura M, Imoto K (2001). A high signal-to-noise Ca^2+^ probe composed of a single green fluorescent protein. Nat Biotechnol.

[pbio-0030355-b26] Heim N, Griesbeck O (2004). Genetically encoded indicators of cellular calcium dynamics based on troponin C and green fluorescent protein. J Biol Chem.

[pbio-0030355-b27] Nagai T, Yamada S, Tominaga T, Ichikawa M, Miyawaki A (2004). Expanded dynamic range of fluorescent indicators for Ca^2+^ by circularly permuted yellow fluorescent proteins. Proc Natl Acad Sci U S A.

[pbio-0030355-b28] Dýez-Garcýa J, Matsushita S, Mutoh H, Nakai J, Ohkura O (2005). Activation of cerebellar parallel fibers monitored in transgenic mice expressing a fluorescent Ca2+ indicator protein. Eur J Neurosci.

[pbio-0030355-b29] Lima SQ, Miesenböck G (2005). Remote control of behavior through genetically targeted photostimulation of neurons. Cell.

[pbio-0030355-b30] Zemelman BV, Lee GA, Ng M, Miesenbock G (2002). Selective photostimulation of genetically ChARGed neurons. Neuron.

[pbio-0030355-b31] Baghart M, Borges K, Isacoff EY, Kramer RH (2004). Light-activated ion channels for remote control of neuronal firing. Nat Neurosci.

[pbio-0030355-b32] Ono T, Nakamura K, Nishijo H, Fukuda M (1986). Hypothalamic neuron involvement in integration of reward, aversion and cue signals. J Neurophysiol.

[pbio-0030355-b33] Harvey MA, Bermejo R, Zeigler HP (2001). Discriminative whisking in the head-fixed rat: Optoelectronic monitoring during tactile detection and discrimination tasks. Somatosens Mot Res.

[pbio-0030355-b34] Kleinfeld D, Sachdev RN, Merchant LM, Jarvis MR, Ebner FF (2002). Adaptive filtering of vibrissa input in motor cortex of rat. Neuron.

[pbio-0030355-b35] Callaway EM (2005). A molecular and genetic arsenal for systems neuroscience. Trends Neurosci.

[pbio-0030355-b36] Schwartz AB (1994). Direct cortical representation of drawing. Science.

[pbio-0030355-b37] Kahana MJ, Sekuler R, Caplan JB, Kirschen M, Madsen JR (1999). Human theta oscillations exhibit task dependence during virtual maze navigation. Nature.

[pbio-0030355-b38] Holscher C, Schnee A, Dahmen H, Setia L, Mallot HA (2005). Rats are able to navigate in virtual environments. J Exp Biol.

[pbio-0030355-b39] Helmchen F, Fee MS, Tank DW, Denk W (2001). A miniature head-mounted two-photon microscope: High-resolution brain imaging in freely moving animals. Neuron.

[pbio-0030355-b40] Zipfel WR, Williams RM, Webb WW (2003). Nonlinear magic: Multiphoton microscopy in the biosciences. Nat Biotechnol.

[pbio-0030355-b41] Denk W, Horstmann H (2004). Serial block-face scanning electron microscopy to reconstruct three-dimensional tissue nanostructure. PLoS Biol.

[pbio-0030355-b42] Tsai PS, Friedman B, Ifarraguerri AI, Thompson BD, Lev-Ram V (2003). All-optical histology using ultrashort laser pulses. Neuron.

[pbio-0030355-b43] Kafkafi N, Benjamini Y, Sakov A, Elmer GI, Golani I (2005). Genotype–environment interactions in mouse behavior: A way out of the problem. Proc Natl Acad Sci U S A.

[pbio-0030355-b44] Miyawaki A, Llopis J, Heim R, McCaffery JM, Adams JA (1997). Fluorescent indicators for Ca^2+^ based on green fluorescent proteins and calmodulin. Nature.

[pbio-0030355-b45] Hasan MT, Friedrich RW, Euler T, Larkum ME, Giese GG (2004). Functional fluorescent Ca^2+^ indicator proteins in transgenic mice under TET control. PLoS Biol.

[pbio-0030355-b46] Baird GS, Zacharias DA, Tsien RY (1999). Circular permutation and receptor insertion within green fluorescent proteins. Proc Natl Acad Sci U S A.

[pbio-0030355-b47] Griesbeck O, Baird GS, Campbell RE, Zacharias DA, Tsien RY (2001). Reducing the environmental sensitivity of yellow fluorescent protein. Mechanism and applications. J Biol Chem.

[pbio-0030355-b48] Pologruto TA, Yasuda R, Svoboda K (2004). Monitoring neural activity and [Ca^2+^] with genetically encoded Ca^2+^ indicators. J Neurosci.

[pbio-0030355-b49] Nagai T, Sawano A, Park ES, Miyawaki A (2001). Circularly permuted green fluorescent proteins engineered to sense Ca^2+^. Proc Natl Acad Sci U S A.

[pbio-0030355-b50] Siegel MS, Isacoff EY (1997). A genetically encoded optical probe of membrane voltage. Neuron.

[pbio-0030355-b51] Guerrero G, Siegel MS, Roska B, Loots E, Isacoff EY (2002). Tuning FlaSh: Redesign of the dynamics, voltage range, color of the genetically encoded optical sensor of membrane potential. Biophys J.

[pbio-0030355-b52] Metzger F, Repunte-Canonigo V, Matsushita S, Akemann W, Diez-Garcia J (2002). Transgenic mice expressing a pH and Cl− sensing yellow-fluorescent protein under the control of a potassium channel promoter. Eur J Neurosci.

[pbio-0030355-b53] Miesenböck G, De Angelis DA, Rothman JE (1998). Visualizing secretion and synaptic transmission with pH-sensitive green fluorescent proteins. Nature.

[pbio-0030355-b54] Boyden ES, Zhang F, Bamberg E, Nagel G, Deisseroth K (2005). Millisecond-timescale, genetically targeted optical control of neural activity. Nat Neurosci.

